# Triple bypass for advanced pancreatic head cancer associated with biliary stricture, duodenal stenosis, and recurrent obstructive pancreatitis

**DOI:** 10.1186/s40792-016-0210-1

**Published:** 2016-08-06

**Authors:** Yuzan Kudo, Norihiro Sato, Toshihisa Tamura, Keiji Hirata

**Affiliations:** Department of Surgery1, School of Medicine, University of Occupational and Environmental Health, Kitakyushu, 807-8555 Japan

**Keywords:** Pancreatic cancer, Palliative, Triple bypass, Obstructive pancreatitis, Pancreaticojejunostomy

## Abstract

Bypass surgery for cancer of the pancreatic head is usually done to palliate the obstructive symptoms in the biliary and/or digestive system. However, it is uncommon for patients to require pancreatic duct drainage for recurrent obstructive pancreatitis. In this article, we report a surgical technique of triple bypass consisting of Roux-en-Y hepaticojejunostomy, gastrojejunostomy, and pancreaticojejunostomy for advanced pancreatic cancer. A 76-year-old male patient with locally advanced and metastatic pancreatic head cancer was referred to our department for biliary stricture, duodenal stenosis, and recurrent obstructive pancreatitis associated with persistent pancreatic pseudocyst. In an attempt to resolve all these problems simultaneously, a triple bypass was performed. The patient survived and continued to receive chemotherapy for almost 1 year after surgery without any serious complications. Thus, triple bypass is a useful surgical technique that could relief symptoms and offer better quality of life to patients with advanced pancreatic cancer presenting with biliary stricture, duodenal stenosis, and severe obstructive pancreatitis difficult to treat by medication or endoscopic procedures.

## Background

Despite an increasing incidence of pancreatic cancer worldwide, the vast majority of patients with pancreatic cancer are diagnosed at an unresectable stage. With new advances in multimodal therapy and resulting increment in the life expectancy of pancreatic cancer, the number of patients with obstructive symptoms of the biliary and gastrointestinal tract could increase. In fact, a recent study has shown that the rate of patients with pancreatic cancer presenting with symptomatic duodenal obstruction has increased [1]. Biliary and/or gastrointestinal bypass, either surgically or endoscopically, is considered to be a choice of treatment for such patients with unresectable pancreatic cancer.

Bypass surgery for pancreatic cancer is usually done to palliate the obstructive symptoms in the biliary and/or digestive system. According to a large-scale analysis of palliative surgery for pancreatic cancer, the bypass surgery performed in 1126 patients included gastrojejunostomy alone (33 %), bile duct bypass alone (27 %), both (31 %), or cholecystojejunostomy (9 %) [2]. In some institutions including our department, a palliative or prophylactic double bypass (Roux-en-Y hepaticocholecystojejunostomy plus gastrojejunostomy with Braun’s anastomosis) was commonly used for patients with unresectable pancreatic cancer. Although obstructive pancreatitis is often seen in patients with pancreatic cancer, it is uncommon for these patients to require pancreatic duct decompression.

In this report, we describe a procedure of bypass surgery, namely triple bypass, to simultaneously resolve the obstruction of the biliary, gastrointestinal, and pancreatic ductal systems. This novel technique can be safely and effectively done in patients with pancreatic head cancer presenting with obstructive jaundice, duodenal stenosis, and severe obstructive pancreatitis.

## Case presentation

**Fig. 1:**
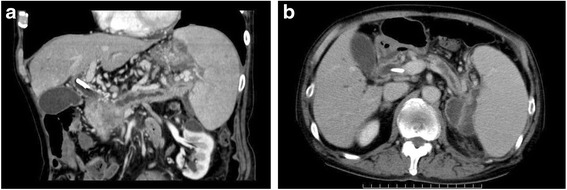
Abdominal CT showing a low-density mass, measuring 40 × 30 mm, in the pancreatic head associated with a marked dilatation of the bile duct and distal pancreatic duct (**a**). There was also an irregular-shaped cystic lesion spreading from the pancreatic tail to the splenic hilum, suggestive of a pancreatic pseudocyst associated with severe obstructive pancreatitis (**b**)

A 76-year-old male patient complaining of appetite loss and upper abdominal pain was referred to our hospital for examination of a mass detected in the pancreas. Laboratory tests showed elevated levels of total and direct bilirubin, amylase and lipase, and CA19-9. Abdominal CT showed a low-density mass, measuring 40 × 30 mm, in the pancreatic head associated with a marked dilatation of the bile duct and distal pancreatic duct (Fig. 1a). The tumor was diagnosed as borderline resectable according to the NCCN guideline based on the finding that it contacted the superior mesenteric vein of >180. There was also an irregular-shaped cystic lesion spreading from the pancreatic tail to the splenic hilum, suggestive of a pancreatic pseudocyst associated with severe obstructive pancreatitis (Fig. 1b). A plastic biliary stent was placed endoscopically, leading to a temporary relief of obstructive jaundice.

During neoadjuvant chemotherapy with gemcitabine and S-1, a follow-up CT at 3 months detected multiple metastases in the liver as well as a metastatic bone tumor in the lumbar vertebrae. The plastic stent was occluded, resulting in a severe cholangitis. Upper gastrointestinal endoscopy showed stenosis of the duodenal lumen due to a projection of the pancreatic head tumor, making it unable to approach the duodenal papilla for stent exchange. The size of pancreatic pseudocyst at the splenic hilum remained unchanged, and endoscopic drainage through EUS was difficult because of its location. The patient also had severe epigastric pain especially after diet resulting from persistent pancreatitis associated with elevated serum levels of amylase (502 U/l) and lipase (1167 U/l). The patient needed to postpone a scheduled chemotherapy for a month because of these symptoms and unsuccessful endoscopic approaches, requiring a bypass surgery. In an attempt to resolve all these problems simultaneously, a triple bypass consisting of Roux-en-Y hepaticojejunostomy, gastrojejunostomy, and pancreaticojejunostomy was performed.

### Surgical procedure

**Fig. 2:**
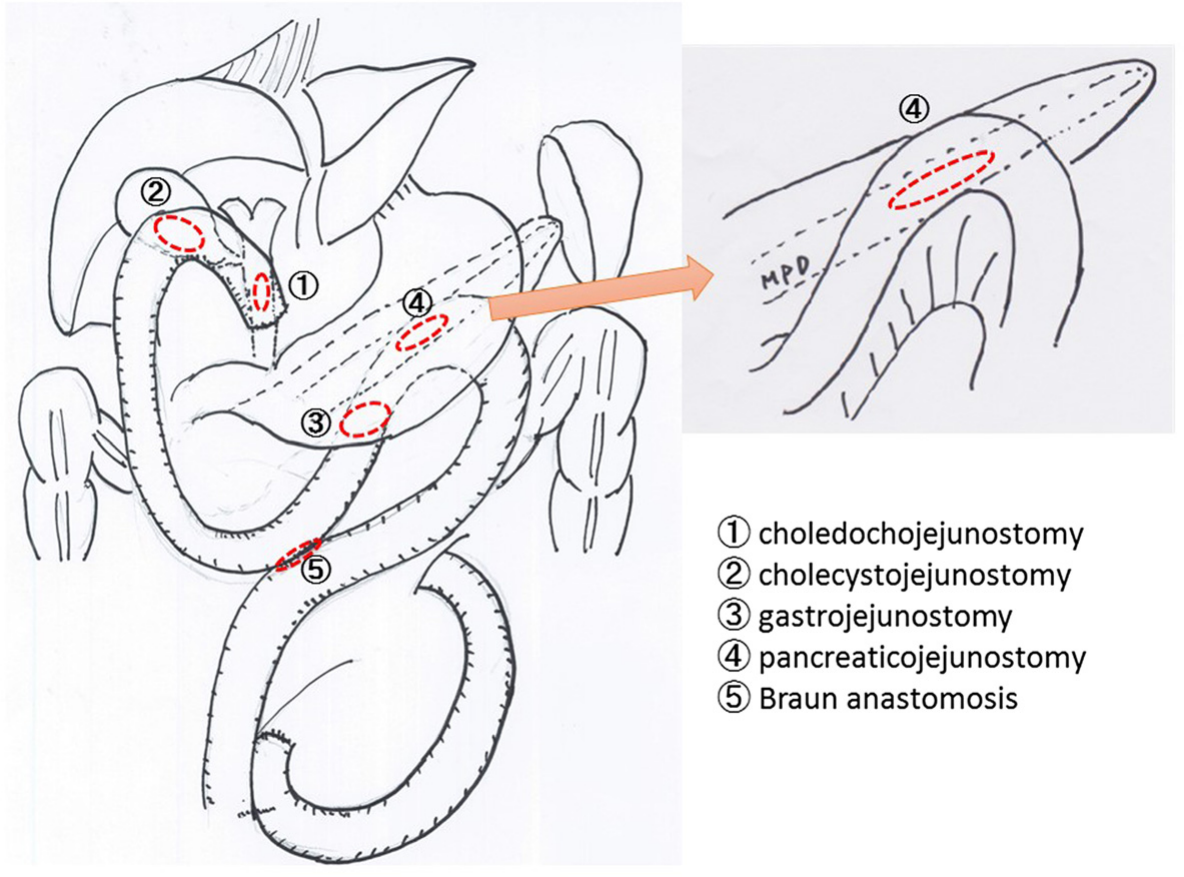
A surgical procedure of triple bypass, consisting choledochojejunostomy (cholecystojejunostomy), gastrojejunostomy, and pancreaticojejunostomy

Under general anesthesia, an upper midline incision was made on the abdomen. A firm mass in the pancreatic head was confirmed, but no peritoneal dissemination was identified. First of all, the proximal jejunum was divided at approximately 15 cm from the Treitz ligament. The divided jejunum was lifted to the hepatic hilum via the antecolic route to make an anastomosis with the dilated common hepatic duct (choledocojejunostomy). We also made cholecystojejunostomy because the cystic duct was also obstructed and the gallbladder remained fully dilated even after biliary drainage. We then lifted the distal jejunum to the posterior gastric wall via the antecolic route to make a gastrojejunostomy. We then dissected the anterior surface of the pancreas and identified the markedly dilated main pancreatic duct using intraoperative ultrasound. After making a 5-cm opening on the main pancreatic duct, we made an anastomosis between the jejunum and pancreatic duct (with interrupted sutures). Finally, a Braun anastomosis was added to complete the triple bypass procedure (Fig. 2).

### Postoperative course

Postoperatively, the patient developed pancreatic fistula, which could be managed conservatively by fasting and drain maintenance. The levels of amylase and lipase decreased to the normal ranges and postoperative CT showed improvements in the pseudocyst in the splenic hilum. The patient was able to restart diet without triggering any abdominal symptoms and was discharged on postoperative day 27. After discharge, the patient immediately started to have chemotherapy with gemcitabine plus S-1 and was able to continue for 11 months, without any signs of biliary obstruction or abdominal fullness. However, at 12 months after bypass surgery, the patient died of pancreatic cancer spreading to the peritoneum, causing bowel obstruction and cachexia.

### Discussion

Despite the recent introduction of endoscopic interventions, bypass surgery remains a treatment of choice for palliating symptoms in patients with advanced pancreatic cancer. Bypass surgery for cancer of the pancreatic head is usually done to relieve the obstructive symptoms in the biliary and/or digestive system. However, it is uncommon for such patients to require pancreatic duct drainage for recurrent obstructive pancreatitis. In this article, we report a surgical technique of triple bypass consisting of Roux-en-Y hepaticojejunostomy, gastrojejunostomy, and pancreaticojejunostomy. Although triple bypass is a complex procedure, this technique can simultaneously resolve the three problems in patients with advanced pancreatic cancer, including obstructive jaundice, duodenal stenosis, and recurrent obstructive pancreatitis. In more specific, we selected triple bypass for this particular case because of the following reasons: (i) Although biliary obstruction in the present case was initially treated by endoscopic stent placement, repeated endoscopic approach to the papilla for stent exchange was subsequently impossible due to exacerbated duodenal stenosis; (ii) The patient complained of nausea and appetite loss possibly due to duodenal stenosis; (iii) The patient suffered from recurrent obstructive pancreatitis associated with pancreatic pseudocyst untreatable by medication or endoscopic procedures.

Recently, endoscopic stent placement for biliary obstruction has been increasingly used as a less invasive alternative to bypass surgery. Recent meta-analysis has shown that endoscopic stenting with plastic stents is associated with a lower risk of complications (RR 0.60, 95 % CI 0.45–0.81) but a higher risk of recurrent biliary obstruction (RR 18.59, 95 % CI 5.33–64.86) than traditional surgical bypass [3]. Distler et al. compared the outcomes of treatment, complications, and survival times between endoscopic stent placement and hepaticojejunostomy for patients with pancreatic head cancer [4]. The authors showed that the mean interval between stent exchanges was 70.8 days because of stent occlusion in patients who were treated with biliary stenting alone. In addition, median survival for patients treated with an endoscopic stenting was significantly shorter than that for patients who were first stented and subsequently treated with hepaticojejunostomy [4]. With recent introduction of more effective chemotherapeutic regimens, including FOLFIRINOX [5] and gemcitabine plus nab-paclitaxel [6], the lifetime of patients with advanced pancreatic cancer could be longer than before. In this regard, bypass surgery or endoscopic placement of stents with a longer patency may be required for patients to benefit these effective chemotherapy.

Obstructive pancreatitis is a common complication in patients with pancreatic head tumor. Although it is slow progressive in most cases, obstructive pancreatic is sometimes associated with severe pain and symptoms as seen in patients with chronic pancreatitis. Previous studies reported treatment of “obstructive” pain by endoscopic pancreatic duct drainage in patients with pancreatic head cancer [7, 8]. However, this endoscopic approach is impossible for patients with duodenal stenosis secondary to a pancreatic tumor. Our approach of pancreaticojejunostomy in addition to the biliary and gastrointestinal bypass could be a treatment of choice for severe obstructive pancreatitis associated with pain.

## Conclusions

In summary, we describe a surgical technique of triple bypass, consisting of Roux-en-Y hepaticojejunostomy, gastrojejunostomy, and pancreaticojejunostomy. This procedure is useful for patients with pancreatic cancer suffering from biliary stricture, duodenal stenosis, and severe obstructive pancreatitis, especially those with a persistent pancreatic pseudocyst untreatable by medication or endoscopic procedures.

## Consent

Written informed consent was obtained from the patient for publication of this case report and any accompanying images.
